# Using the Bayley-4 and Vineland-3 in Angelman syndrome: barriers, solutions, and challenging items

**DOI:** 10.1186/s13023-025-03817-x

**Published:** 2025-06-03

**Authors:** Sean N. Halpin, Sarah Nelson Potter, Angela Gwaltney, Anjali Sadhwani, Katherine C. Okoniewski, Anne C. Wheeler

**Affiliations:** 1 GenOmics and Translational Research Center, RTI International, 3040 East Cornwallis Road, Research Triangle Park, Durham, NC 27709-2194 USA; 2 Department of Psychiatry and Behavioral Sciences, Boston Children’s Hospital, Boston, MA USA

**Keywords:** Angelman syndrome, Assessment, Neurodevelopmental disorders, Rare diseases

## Abstract

**Objective:**

The Bayley Scales of Infant and Toddler Development- 4th Edition (Bayley-4) and Vineland Adaptive Behavior Scales – 3rd Edition (Vineland-3) are outcome measures often considered as primary endpoints in clinical trials for Angelman syndrome (AS). We explored barriers encountered when administering these instruments to individuals with AS and associated guidance for their use in trials and research studies.

**Methods:**

We interviewed nine clinicians who have administered the Bayley-4 and/or the Vineland-3 to individuals with AS and analyzed their transcripts using a quasi-deductive analysis approach.

**Results:**

Barriers to administering the Bayley-4 included participant’s lack of interest, overexcitement, emotional impact on caregiver, the mental workload of administering the Bayley-4, and environmental factors (e.g., administration setting). Barriers to administering the Vineland-3 included determining the most appropriate start point, emotional impact on caregiver, distractions, conflicting answers from two caregivers, and the mental workload of administering the Vineland-3. Participants provided potential solutions to each barrier. Lastly, we identified overarching item-level concerns for both the Bayley-4 (i.e., administration challenges, items not aligned with abilities) and the Vineland-3 (i.e., misalignment of assessment criteria and condition characteristics, limitations in observation and contextual understanding, requires specialized training).

**Conclusion:**

Clinical trials often rely on the Bayley-4 and Vineland-3 assessments as outcome measures, yet our identified barriers threaten their validity. The associated solutions provide a path forward for improving administration of the Bayley-4 and Vineland-3 in clinical practice, research, and future trials focused on individuals with AS and other intellectual and developmental disabilities.

**Supplementary Information:**

The online version contains supplementary material available at 10.1186/s13023-025-03817-x.

## Introduction

Angelman syndrome (AS) is a rare neurogenetic condition with prevalence estimates ranging from 1 in 10,000 to 1 in 20,000 [1–3]. AS is characterized by severe to profound intellectual disability (ID), seizures, sleep disturbances, ataxia, a distinctive behavior profile (e.g., frequent smiling, laughing, overexcitement, anxiety), and significantly limited verbal communication such that most individuals with AS are nonspeaking [4]. Many promising disease-modifying treatments exist in the preclinical and clinical phases of development for AS, as well as ongoing phase I/II trials that are testing their safety, side effects, best dose, and efficacy (NCT04259281, NCT05011851, NCT05127226, NCT05630066) [5]. Because these treatments are hypothesized to be disease-modifying, measures that capture global change across multiple domains of functioning have been deemed most appropriate. However, identifying outcome measures that capture change in cognition and daily functioning, and are valid and reliable for use in AS has been a significant challenge [6].

Traditional cognitive measures are often unsuitable for AS due to multiple concerns, including floor effects, as individuals cannot complete enough items for valid scores [7–9]. Standardized assessments also pose challenges in other populations with severe developmental impairments [10]. As a result, most clinical research, including the nearly two decades long natural history study for AS, has used the Bayley Scales of Infant and Toddler Development (Bayley) [11], as the core measure of development and cognition [7–9, 12–14]. The Bayley is a direct assessment of five domains of development—Cognition, Receptive Language, Expressive Language, Fine Motor and Gross Motor. Items on the Bayley were designed to assess emerging developmental milestones typically obtained in the first three and a half years of life. Because the Bayley was developed to be administered to newborns (i.e., as early as 16 days after birth), all individuals with AS can complete at least some of the items [9].

However, administering the Bayley to individuals with AS out of age norms poses multiple challenges, including that many items are not developmentally appropriate for older children and adults, which may lead to a lack of attention or motivation in the child, and be upsetting for caregivers [9]. Other limitations include that the Expressive Language subtest relies on vocalizations or verbal communication. Because nearly all individuals with AS are nonspeaking and may use alternative modes of communication (e.g., augmentative and alternative communication [AAC] systems), the Bayley may not capture their expressive communication skills because it relies predominantly on oral communication skills.

Clinical studies usually supplement the Bayley with the Vineland Adaptive Behavior Scales (Vineland) [7, 8, 12, 14–17], which provide a caregiver perspective on developmental and daily living skills through a structured interview [11, 18–23]. The Vineland Adaptive Behavior Scales, Third Edition (Vineland-3) is the most recent edition of the Vineland, and measures communication (i.e., receptive, expressive, written), daily living skills (i.e., personal, domestic, community), socialization (i.e., interpersonal relationships, play and leisure, coping skills), motor skills (i.e., gross motor, fine motor), and maladaptive behavior (i.e., internalizing behavior, externalizing behavior) [23]. Although a caregiver report form is available, the Vineland-3 is typically administered as a comprehensive interview with a caregiver, and it has been used to assess adaptive functioning in AS in several studies, including in clinical trials and longitudinally in the Angleman Syndrome Natural History Study, providing in-depth understanding about adaptive functioning over time in AS [7, 8, 12, 14–17]. The Vineland-3 was developed to assess adaptive behavior across the lifespan (i.e., from birth to 90 years of age), and unlike the Bayley-4 which captures performance on a single day, the Vineland-3 assesses what an individual can do on a daily basis across a wide range of skills and activities of daily living. However, items are subject to interpretation by both the caregiver and the examiner, and the reliability of parent report depends on multiple factors (e.g., under- vs. overestimation of skills, familiarity with developmental milestones) [24, 25].

Given the reliance on the Bayley and Vineland to assess development in individuals with AS and the likelihood of their inclusion as endpoints in upcoming clinical trials, identifying ways to improve their use in research studies and clinical trials is of upmost importance. These assessments have been used to evaluate cognitive and adaptive functioning in AS [7–9] particularly in longitudinal studies and trials. However, no prior research has systematically examined administration challenges or proposed solutions in AS. This study addresses that gap by identifying key barriers and strategies to enhance administration in research and clinical settings. In the current study, we sought to identify challenges experienced by assessors during the administration of the Bayley-4 and Vineland-3 to individuals with AS and develop strategies to address identified barriers. These strategies provide guidance for improving the use of these assessments in future research studies and clinical trials.

## Methods

### Overview

The current study is part of a larger effort to identify weakness and barriers in the administrations of the Bayley-4 and Vineland-3 with the goal of developing standardized processes for improving the use of these tools in AS clinical trials. This study involved qualitative interviews with clinicians who have administered the Bayley-4 and Vineland-3 in individuals with AS with the purpose of understanding challenges in administration and scoring of these assessments and strategies implemented to address these challenges [26]. RTI’s Institutional Review Board reviewed and approved the study. We obtained verbal informed consent from all participants before beginning the interviews.

### Data collection

We recruited interview participants using criterion and snowball purposeful sampling [27], selecting clinicians with direct experience administering the Bayley-4 and/or Vineland-3 to individuals with AS. Given the rarity of AS and the specialized nature of these assessments, the pool of qualified assessors is small. However, our participants represented diverse geographic locations (United States, Australia, United Kingdom, Canada) and had substantial experience in research and clinical trials. Although the sample size was limited, qualitative research prioritizes conceptual rather than statistical generalizability. We observed saturation of key themes, indicating that core challenges and solutions were well-captured. Clinicians’ backgrounds likely influenced the perspectives shared in interviews. Those with clinical trial experience were familiar with adapting assessments to structured research protocols, while those working in community-based settings described different challenges, such as variable caregiver engagement and resource constraints. These diverse professional contexts provided complementary insights into the real-world administration of the Bayley-4 and Vineland-3, strengthening the applicability of our findings to both research and clinical practice.

First, authors AS and ACW identified clinicians who administered the Bayley-4 and Vineland-3 to individuals with AS in research and clinic settings, prioritizing those who had experience administering one or both of these measures in AS Phase II clinical trials. One participant recommended an additional expert who we later interviewed. SNH contacted and scheduled participants. Participants completed an online pre-interview survey using a provided link. The REDCap (Research Electronic Data Capture) survey (Appendix A.) included questions regarding demographic information (5 items), education and training (4 items), and experience with the Bayley and Vineland assessments (15 items) [28, 29]. REDCap is a secure, web-based software platform designed to support data capture for research studies, SNH conducted all interviews using the Microsoft Teams virtual meeting platform and a semi-structured interview guide (Appendix B.) [30]. The full team developed the semi-structured interview guide including authors with expertise in AS (AS, ACW, SNP, KCO, AG) and administering the Bayley-4 and Vineland-3 (AS, ACW, KCO) and an author with qualitative methods expertise (SNH). SNH tailored the interview based on the answered pre-interview questions (e.g., What section of the Bayley Scales are most challenging to administer?). Further, SNH used the screen share function to review item from the Bayley-4 and Vineland-3 record forms during the interview. We audio recorded and transcribed verbatim all interviews using the Microsoft Teams transcription tool and reviewed the transcripts for accuracy.

### Data analysis

We used NVivo version 14.0 (Lumivero, United States) for data management and analysis and applied a quasi-deductive analysis approach, which combines structured coding with flexibility to capture emergent themes [31]. We began with a deductive framework, developing an initial codebook based on our interview guide topics, such as “barriers to assessment” and “caregiver emotional response” (Appendix B.). At the same time, we allowed for inductive coding, identifying new themes that arose organically in clinician responses. For example, while we expected clinicians to report difficulty determining the correct Vineland-3 starting point, we did not anticipate challenges related to caregiver disagreements during the interview. This blend of deductive structure and inductive discovery ensured a comprehensive understanding of clinician experiences. Interview recruitment ceased once no new concepts emerged from subsequent interviews. SNH and SNP independently coded all transcripts. Discrepancies were resolved through consensus coding, which involved reviewing participant text and discussing differences with the larger group. The full author team met weekly to review coding matrices and support interpretation of emergent themes. All team members reviewed the associated data and coding, and matrix groupings and verified attributed quotes for Bayley-4 and Vineland-3 items.

### Analytical rigor

We assured analytical rigor through triangulation and member checking [32, 33]. We applied triangulation of sources through the purposeful sampling of experts with administration experiences across diverse geographical countries. Further, we achieved analyst triangulation through the data interrogating and interpretation by all authors. We also employed member checking with participants, iteratively clarifying emergent results with participants [31].

## Results

### Participants

Nine assessors who administered the Bayley and or Vineland to individuals with AS were interviewed. The interviews had a median length of 91 min (IQR = 72–122 min). Participants had a median age of 38 (IQR = 28–47), were mostly female (*n* = 7, 78%), white (*n* = 8, 89%), and had a master’s or doctoral degree (*n* = 8, 89%) (Table 1.). Our participants resided in the United States (*n* = 5, 56%), Australia (*n* = 2, 22%), United Kingdom, (*n* = 1, 11%), and Canada (*n* = 1, 11%). Participants who administered the Bayley-4 (*n* = 9, 100%) had a median of eight years (IQR = 2–23) experience administering the assessment. The majority indicated their training included reviewing the Bayley-4 manual (*n* = 7, 78%). Five (56%) indicated they observed videos of others performing the assessment. Other training activities included research training (*n* = 2, 22%), in-person observation (*n* = 2, 22%), dyadic training (*n* = 2, 22%), and the provided Pearson training (*n* = 2, 22%). As shown in Table 1, the Cognitive Scale was most frequently identified as the most challenging Bayley-4 section to administer (*n* = 7, 78%), followed by Receptive Communication Subtest (*n* = 4, 44%). Participants were allowed to report more than one section as most challenging, which accounts for totals exceeding 100%. Those who administered the Vineland-3 (*n* = 6, 67%), had a median of nine years (IQR = 3–23) administration experience. Training procedures for the Vineland-3 included reviewing the manual (*n* = 4, 67%), observing videos of others performing the assessment (*n* = 3, 50%), in-person observation (*n* = 1, 17%), and the provided Pearson training (*n* = 1, 17%). The most frequently cited challenges for the Vineland-3 included the Maladaptive Behavior domain, with internalizing, externalizing, and critical items each reported by 67% of participants (see Table 1).

**Table 1:** Participant Demographic Characteristics

Characteristic	All Participant (*n* = 9) *	Participants who administered the Vinland-3 (*n* = 6)
Median age (years)	38 (IQR = 28–47)	42 (IQR = 28–47)
Female, n (%)	7 (78%)	4 (67%)
Race		
Asian, White	1 (11%)	1 (17%)
White	8 (89%)	5 (83%)
Education		
Bachelor’s degree	1 (11%)	0 (0%)
Master’s degree	5 (56%)	3 (50%)
Doctoral degree	3 (33%)	3 (50%)
Country of Residence		
Australia	2 (22%)	1 (17%)
Canada	1 (11%)	0 (0%)
United Kingdom	1 (11%)	0 (0%)
United States	5 (56%)	4 (67%)
Years working with people with neurogenetic/neurodevelopmental disabilities		
In clinical/healthcare	15 (IQR = 0–25)	16 (IQR = 2–25)
In clinical trials	7 (IQR = 2–15)	6 (IQR = 2–15)
In non-clinical trials	12 (IQR = 2–25)	11 (IQR = 2–20)
Years spent researching individuals with AS		
In clinical/healthcare	7 (IQR = 0–12)	8 (IQR = 2–10)
In clinical trials	2 (IQR = 2–10)	3 (IQR = 2–10)
In non-clinical trials	2 (IQR = 0–12)	1 (IQR = 0–10)
Age of AS Population you have worked with		
Lowest age (months)	36 (IQR = 6–60)	36 (IQR = 12–48)
Highest age (years)	25 (IQR = 17–48)	25 (IQR = 17–45)
Context administering the Bayley		
In clinical/healthcare	7 (78%)	5 (83%)
In clinical trials	8 (89%)	6 (100%)
In non-clinical trials	6 (67%)	4 (67%)
Most challenging Bayley section to administer#		
Cognitive Scale	7 (78%)	5 (83%)
Language Scale- Receptive Communication Subset	4 (44%)	3 (50%)
Language Scale- Expressive Communication Subset	1 (11%)	1 (17%)
Motor- Fine Motor Subset	1 (11%)	1 (17%)
Motor- Gross Motor Subset	1 (11%)	0 (0%)
Context administering the Vineland		
In clinical/healthcare	3 (67%)	4 (67%)
In clinical trials	5 (100%)	6 (100%)
In non-clinical trials	4 (67%)	4 (67%)
Most challenging Vineland section to administer#		
Maladaptive Behavior Domain- Internalizing	4 (67%)	4 (67%)
Maladaptive Behavior Domain- Externalizing	4 (67%)	4 (67%)
Maladaptive Behavior Domain- Critical Items	4 (67%)	4 (67%)
Communication Domain- Receptive	1 (11%)	1 (11%)
Daily Living Skills Domain- Community	1 (11%)	1 (11%)
Socialization Domain- Play and Leisure	1 (11%)	1 (11%)
Socialization Domain- Coping Skills	1 (11%)	1 (11%)

* all participants with experience administering the Bayley-4 also administered the Vineland-3

# Participants could report more than one section as most challenging; therefore, totals may exceed 100%

### Barriers and solutions to administering the Bayley-4 to individuals with Angelman syndrome

Five barriers were identified in the administration of the Bayley related to either the individual with AS (lack of interest, reduced motivation, being overexcited); the caregiver (emotional response) or the examiner (mental workload). For each of these barriers, participants were asked to provide possible solutions. All barriers and solutions are presented in Table 2.

**Table 2:** Bayley-4 Barriers and Solutions to Administer to Persons Living with Angelman Syndrome

Barrier	Barrier Description	Solution	Solution Description
Bayley-4 Participant Barrier A: Lack of Interest and Reduced Motivation	All participants (*n* = 9, 100%) identified a lack of interest and reduced motivation of the individual with AS as a barrier to administering the Bayley-4 in this population. For example, one participant discussed the differences between neurotypical children and individuals with AS, “If you take a neurotypical child, even that’s like two-years old, there’s enough of a desire and a motivation to please the adult or please the rater and a lot of cases that they’ll stay engaged with you however long. In Angelman syndrome…you really start having to fight for it [their attention]” (PT6).	Bayley-4 Participant Solution A1: Punctuality	All participants (*n* = 9, 100%) described the importance of punctuality for engaging the participant and maintaining their interest. One participant stated, “when our parents rock up, they’ll go straight into our car park, we’ll go and meet them downstairs in the service lift, we’ll zip them straight up into our clinic room” (PT4).
		Bayley-4 Participant Solution A2: Engage Caregivers	Over half of participants (*n* = 6, 67%) discussed how engaging with the parents could combat a lack of interest and reduced motivation. Prior to the assessment, the assessor would ask the caregiver whether there were any motivators they should be aware of or anything to avoid during the Bayley-4; “So I start with a very calm demeanor and I ask, are there any things specifically I need to know about your kid?…So, some of them like don’t like it when you touch their hand for example” (PT3).
		Bayley-4 Participant Solution A3: Switch to a More Engaging Section	Nearly half of participants (*n* = 4, 44%) would switch to a more engaging section as a strategy for maintaining the participants interest, especially the motor section. When asked whether she takes breaks, one participant answered, “I would say if needed, but one of the breaks I kind of tend to work in is getting some of the gross motor done. So especially if I can just tell they need to wiggle…I love to do like a gross motor break.” (PT9).
		Bayley-4 Participant Solution A4: Spotlight on Administrator	A third of participants (*n* = 3, 33%) discussed the need to “be the most interesting thing in the space” in order to keep the participants interest. One participant explained, “there are times where I just…I’ll knock something on the on the desk just to get their attention. You know, just to make that a little more enticing and a little more exciting to engage with” (PT6).
Participant Barrier B: Emotional Impact on Caregiver	Some participants (n = 4, 44%) reflected that caregivers may become sad when observing tasks their child had difficulty completing, leading to apprehension of asking certain questions. Parents’ reactions ranged from physical displays of sadness (e.g., crying) to attempts to intervene during the assessment and influence how their loved one would answer items. One participant stated “so what ends up happening is that these parents feel really discouraged because this test was designed for neurotypical kids, not for their children. And their kids cannot do a lot of these items” (PT3).	Participant Solution B1: Being Empathetic	A solution to the emotional impact on caregivers was being empathetic, assuring, and explaining what to expect with the test ahead of time (*n* = 4, 44%). One participant stated, “I explain what the Bayley is like. These are the different domains is how long it’s going to take…and then we also talk about how sort of like the point before, there’s likely things that your child can do at home that I’m not gonna see…and I’m gonna ask a different way and it’s no judgement. It’s just this is how they do it in this on this particular day with these particular materials and this particular instructions so that we can compare, we do it the exact way every time” (PT1).
Participant Barrier C: Participant Overexcited	Three participants (33%) discussed the individual with AS being “overexcited” as a barrier to administering the Bayley-4. Being overexcited was in stark contrast to participants who lacked interest and had reduced motivation. This barrier was exemplified by one participant who stated, “some of the parents will say that they’ll [the person with AS] will get excited when they start getting closer [to the testing location]” ( PT3). As a result, overexcited participants would fixate on objects and have difficulty completing the required Bayley-4 tasks.	Participant Solution C1: Switch to a Calm Demeanor	A minority of participants (*n* = 3, 33%) talked about the need to switch to a calm demeanor to address when a person living with Angelman syndrome become over excited and not focused on the administration. Here a participant stated, “now it’s work time and get a little more flat affect…a lot of what I do, I feel like is like reading the room, you know, like read the kid, read the mom, ask mom” (PT9).
Assessor Barrier D: Mental Workload of Bayley-4	Over half of participants (*n* = 6, 67%) discussed the mental workload of administering the Bayley-4, including the need to efficiently access the manipulatives associated with many items. As one participant explained, “[its about] getting really fast with the materials and knowing exactly what they were” (PT01). Further, the mental workload was exacerbated when administering the Bayley-4 to an individual with AS.	Assessor Solution D1: Familiarity with Materials	Concerning the cognitive demand of administering the Bayley-4, participants discussed the importance of the administrator being very familiar with the materials (*n* = 6, 67%). As one participant stated, “you have to know that you can’t be reading it and figuring it out [during the assessment]” (PT7).
		Assessor Solution D2: Know the Purpose of Each Item	Two participants (22%) discussed the value of knowing the purpose of each item. One participant stated, “You know, a newer rater or somebody with less experience, or someone who’s really not thinking. Oh wait, it’s the what is? What section am I in and to say Please remember for this it is important that they know that a spoon is a spoon. Not that it that it’s something you eat with or don’t say. Where’s the Clifford thing? Because then they reach for the book. You know you you have to. Yeah, I mean, honestly, that that’s an interesting concept to think like, you know, the purpose of this item is fine motor if you have to call the Cheerios Spooky Wookies and ask them to pick them up. It doesn’t matter. It’s about their pincer grasp” (PT9).
Environmental Factors Barrier E	All participants (*n* = 9, 100%) endorsed environmental factors, including whether the individual with AS was tired from travel and/or recent medical treatments, that could negatively affect their ability to engage. One participant stated, “just that subtle difference in their routine can have an impact on their attention span and all that kind of stuff. So we actually really notice a difference when the family does travel in the day prior and then get a good night’s rest” (PT6). The testing environment was also an external barrier, as distracting items in the room could affect the concentration or performance of the individual being assessed. As stated by one participant, “There’s no like cat posters, and you know those kind of things? Umm, I think some small or muted items could be OK, but I I wouldn’t say you want a lot of distraction where they’re like looking at the kitty cats all the time or whatever” (PT9).	Environmental Factors Solution E1: Consider Ideal Timing	All participants (*n* = 9, 100%) stated it was necessary to consider ideal time of day for administering the test, when the participant would be at their best. A participant stated, “I’m a big believer that the parent is always the expert when it comes to their child. So if the parent’s sitting there saying they don’t perform well first thing in the morning, they’re better after lunch person. And then we’ll we’ll we’ll schedule in something like this for after lunch. So, we let the parents really dictate how we sort of planned the day” (PT4). Other participants felt mornings were always the better time to perform the Bayley-4, especially before the participant was involved in other assessments that day, “I am the one who sees the patient first in the morning. So I do the Bayley, that’s for sure. Doing the Bayley in the afternoon is is is is a lost cause” (PT2).
		Environmental Factors Solution E2: Decrease Environmental Distractions	A majority (*n* = 7, 78%) discussed the need to decrease environmental distractions–as stated by one participant “you know there’s a lot of times that in pediatric clinics, there’s stuff on the walls and and stuff, you know, there just stuff everywhere and that can be actually quite distracting to this population. So we’ve like plain, boring, dull white, you know, a little more clinical to be honest, just because it’s that much less of a distraction” (PT6).
		Environmental Factors Solution E3: Setup Room Ahead of Time	Participants (*n* = 6, 67%) also discussed the need to set-up the room ahead of time, including having their Bayley-4 kit arranged and ready. One participant stated, “we’re really, ummm, deliberate and careful about our preparation. So you know, we set up the room in advance” (PT1).

### Bayley-4: challenging domains to administer to individuals with Angelman syndrome

Nearly all participants (*n* = 8, 89%) felt the Cognitive Domain was the most challenging Bayley-4 domain to administer followed by Receptive Communication (*n* = 6, 67%), Fine Motor, (*n* = 5, 56%), and Gross Motor (*n* = 1, 11%). Overall, participants felt the Bayley assessment was not appropriate for the wide age range of individuals with AS that receive this assessment as part of a research study or clinical trial protocol. One participant explained, “this comes down to the limitations of administering a test that’s meant for like, this is like an 8-month-old level versus like an 8-year-old. Some of the things that they’re experiencing that they’re interested in are just beyond what we’re giving them in this test” (PT3). Themes related to challenges in administering specific items are presented in Table 3.

**Table 3:** Bayley-4 Challenging Items to Administer to Persons Living with Angelman Syndrome

Overview	Themes	Description
Administration Challenges	Practical limitations in assessment procedures	Nearly half (*n* = 4, 44%) of participants described practical limitations to administering the Bayley-4 in adults with AS. For example, one participant talked about a question requiring the assessor to hold the participant in a prone position, stating “Then you will arrive at where a child needs to be able, where you check and you see how the patient is actually keeping his head aligned with the body while you are elevating it. And it’s just not possible. You cannot assess that. So what do you do? Do you you you use your good clinical judgment and you’re like OK patient is able to keep a sitting positions. So for me, I think it’s fine. He keep his leg head aligned. Yeah. Or with child is for example completely supported. You see, he has no head support. Then you know that you will be able to score this zero. But but those are again those limitations” (PT2).
Items Not Aligned with Abilities	Engagment and accessibility of assessment tools	Four (44%) of participants discussed how the assessments associated manipulatives are not engaging and accessible for people living with AS. One participant stated, “I would actually say that that’s what I think is kind of hard about things like the Bayley is nothing has moved into being electronics. So when you go to show them the receptive or expressive or whatever items that are just in this flip book that’s not laminated and it has these coils that they love and get very distracted by every single time, umm they want to rip it or they’re not interested or they don’t seem to point in the parent or, you know, and they can pat if they want to register and the parent is like, oh, if this was on a tablet because that is enticing to them and that is how they work with either their AAC” (PT9).
Items Not Aligned with Abilities	Scoring progression is not aligned with AS abilities	About a quarter of participant (*n* = 2) worried that the scoring progression was not aligned with the abilities of someone living with AS. One participant stated, “So, and then you have here item 30 rises self to standing. So the patient is ambulatory now, is able to raise himself from 2 standing by pulling up on furniture. That’s great. Then you’re if you’re walking, it’s takes at least four steps with support, with good coordination. Almost never happens because none of those kids has a good coordination. So basically you will have to go back” (PT2).

### Barriers to administering the Vineland-3 to caregivers of individuals with Angelman syndrome

We identified six barriers to administering the Vineland-3: two participant barriers (Emotional Impact on Caregiver and Conflicting Answers from Two Caregivers); and four assessor barriers (Determining Start Point; Determining Best Answer; Managing Distractions; and the Mental Workload of Vineland-3 on the Assessor). All barriers and solutions are presented in Table 4.

**Table 4:** Vineland-3 Barriers and Solutions to Administer to Caregivers of Loved Ones with Angelman Syndrome

Barrier	Barrier Description	Solution	Solution Description
Vineland-3 Participant Barrier A: Emotional Impact on Caregiver	Most participants (*n* = 5, 83%) stated the emotional impact on the caregiver was a barrier to administering the Vineland-3. A participant explained why they felt caregivers were more likely to become emotional during the Vineland-3 compared to the Bayley-4: “Yeah, I I it’s funny because parents can be totally different between the Bayley and the Vineland uh. And what I mean by that is like when the child’s there and you’re trying to pull everything out of the child, the parent…. they’re just trying to control their child [and don’t have time to process how well their child is doing on the assessment]. Whereas when you really get them away from their child, I think that the Vineland is much more reflective [of their child’s abilities]. And it really does allow them to just sit and think about the good things that their child can do. And sometimes, unfortunately, the things that their child can’t do” (PT6)	Vineland-3 Participant Solution A1: Being Empathetic	Being empathetic was a key solution to handling the emotional impact on caregivers for most participants (*n* = 5, 83%). One participant explained, “I try and sort of yeah. Put that clinical hat on and take the researcher hat off and just sort of say, yeah, you know, this is incredibly hard. I wish that I could do this, you know, a different way. So, it could be a bit more palatable, you know, let’s take a couple of minutes…nine times out of 10, the parents will, you know, have a bit of a cry” (PT4).
		Vineland-3 Assessor Solution A2: Consider Items Covered Previously	At times topics would generically come up that answered future questions, and participants discussed the importance of their remembering these topics; “So I try to even if I can’t go straight to it just later, just say like you mentioned, he loves running up the steps. Tell me more about that. um, don’t pretend like you haven’t already heard all these things” (PT9).
Participant Barrier B: Conflicting Answers from Two Caregivers	Half of the participants (*n* = 3, 50%) described two caregivers providing conflicting answers as a barrier. One participant stated, “it happens often enough that they do initially have different answers” (PT6).	Participant Solution B1: Talk it Out	Often participants would come to consensus by simply “talking it out” between two caregivers who gave conflicting answers. This was explemplified by a participant who stated, “I usually say, OK, can we agree that this is what is going to be the best score here and both of them then agree” (PT6).
		Participant Solution B2: Pick One Parent	One participant (*n* = 1, 17%) periodically had to make a judgement on which parent’s answer they would report, stating “90% of the time they’re in agreement, the 10% they’re not. I have to pick one parent in my head” (PT9).
Assessor Barrier C: Determining Start Point	All participants who administered the Vineland-3 (*n* = 6, 100%) suggested that finding the start point was a barrier. One participant elaborated on the receptive communication domain, “it depends on the kid, right? Like so, if it’s the kid is really impaired, I often will start with like, how do you get his attention…or if it’s a higher functioning kid, I might say [to the caregiver] ‘what words does he understand? How do you communicate with him? I don’t know. It really depends on the child. So, I sort of start really really really broad” (PT1).	Assessor Solution C1: Review Previous Vineland Administrations	Over half of participants (*n* = 4, 67%) stated that they reviewed previous Vineland administrations to help them determine a starting point. Participants were cautious not to “prefill in anything and assume that they’re still doing that” but also considered the importance of being contentious, “I do like to look at old ones if the study allows it, just so I don’t start and sound like again like I’ve never talked to you before. I don’t want to come across like I don’t remember that we met and we talked for an hour and you told me all these things about your kid. I don’t wanna act like that is nowhere in my brain, so I always go” (PT9).
		Assessor Solution C2: Based on Observation	Participants (*n* = 4, 67%) also based their starting point on any chance they had to observe the child before administering the Vineland-3, including if they completed the Bayley-4 first. One participant stated, “I think it’s a little easier when you’ve just been with the kid” (PT9).
		Assessor Solution C3: Start at the Beginning	One participant (*n* = 1, 17%) preferred starting at the beginning, a strategy that was emotionally more satisfying for the caregiver than starting immediately with items their loved one could not complete. This participant stated, “cause that feels really good as opposed to starting at like, you know, a different starting point and then they have to answer no to a lot of items right off the bat” (PT5).
Assessor Barrier D: Determining Best Answer	All participants (n = 6, 100%) stated determining the best answer was a barrier—as one participant explained their strategy for probing to ensure their answer is as accurate as possible, for example an item assessing how long a participant can attend to a show; “ [the parent says] he was watching a YouTube video and I think it was about ten-minutes but I cannot be sure…OK well, what were you doing in that ten minutes?” (PT5). The participant went on to describe a strategy for measuring time against other activities the participant was engaged with during that time, like going to the bathroom, and asking whether the parent was in the bathroom for ten-minutes.	Assessor Solution D1: Additional probing	Additional probing helped some participants (*n* = 2, 33%) to determine the best answer. As exemplified by one participant who described the process they used to help a parent determine the amount of time the child participated in an activity, “I was like ohh, OK well, what were you doing in that 10 minutes to think it was a 10-minute block and then she might be like mum might be like Oh well, I went to the bathroom and came back. I was like, well, would you typically spend 10 minutes going to the bathroom or not? Really. I was like, well, maybe that’s giving us a bit of an indication that it was less than 10 minutes. So, for this particular item, where it’s 15 minutes, we’re probably gonna go and never and then just move on to the next one” (PT4).
		Assessor Solution D2: Assessors Judgement	One participant (*n* = 1, 17%) discussed the rare instance when the assessor would make a judgement on what score to report, stating " I’ve worked with the kid. I know what’s typically rated. You know, there’s no way we’ve jumped 20 points in that span of, you know, or something like that. So I feel like you have to have some discretion with that” (PT5).
Assessor Barrier E: Distractions	Some participants (*n* = 4, 67%) stated that distractions constituted a barrier both when the Vineland-3 is administered in the clinic and when it is done remotely. Distractions were often related to childcare, as one participant stated, “just the normal family interruptions” (PT9).	Assessor Solution E1: Plan Caregiving During Administration	Over half of participants (*n* = 5, 67%) discussed the need to plan caregiving during Vineland-3 administration to avoid distractions. One participant stated, “maybe they’ve organized for an uncle or auntie to come around just to give moms and dads you know that free hour, just to, you know, I guess, concentrate on one thing” (PT4).
Assessor Barrier F: Mental Workload of Vineland-3 on the Assessor	Two participants (*n* = 2, 33%) felt the mental workload of administering the Vineland-3 was a barrier especially as it relates to ensuring they remain attentive to the caregiver and not miss any items. A participant explained, “I messed up before, but like I am really, really careful to make sure I don’t miss an item” (PT1).	Assessor Solution F1: Take Time to Review Answers	Two participants (*n* = 2, 33%) addressed the cognitive demands of administering the Vineland-3 by taking time to review the answers. One participant explained, " I check at the end of the interview I actually like, I always tell the caregiver just a moment. I’m gonna make sure I did them all and then I go back page by page” (PT1).

### Vineland-3: challenging domains to administer to caregivers of individuals with Angelman syndrome

Over half of participants (n = 4, 67%) felt the Maladaptive Behavior domain was inappropriate for administering to caregivers of individuals with AS, especially items having to do with masturbation, violence, and suicide. One participant explained:

“I don’t want to have to ask these parents questions that seem like you’re not understanding me or you’re not understanding my child, or you don’t understand what Angelman’s is because I think that’s often a big thing for the Angelman’s community or any genetic syndrome.” (PT8).

Participants did not have concordance on other challenging sections, with just one participant (*n* = 1, 11%) stating challenges existed for each of the following subdomains: Receptive Communication, Community, Play and Leisure, and Coping Skills. Themes related to challenging Vineland-3 items are presented in Table 5.

**Table 5:** Vineland-3 challenging items to administer to caregivers of loved ones with Angelman syndrome

Themes	Description
Misalignment of Assessment Criteria and Condition Characteristics	A third of participants (*n* = 3) talked about how the Vineland-3 included questions that were inappropriate for persons living with AS. One participant explained, “a lot of the things are just not applicable. I mean it’s like. Has thoughts of suicide or, you know, bullies, others and things that are just, I mean, yeah. I mean, there’s quite a few. Like how has seems to have ideas that are not real or, you know, seems to have a. How would the parent know? Umm. And then there’s some that it’s it’s like, well, yeah, they do that, but it’s because of their disorder” (PT9).
Limitations in Observation and Contextual understanding	Two participants (22%) worried about the accuracy of parents responses, with one stating “a lot of the things are just not applicable. I mean it’s like. Has thoughts of suicide or, you know, bullies, others and things that are just, I mean, yeah. I mean, there’s quite a few. Like how has seems to have ideas that are not real or, you know, seems to have a. How would the parent know? Umm. And then there’s some that it’s it’s like, well, yeah, they do that, but it’s because of their disorder” (PT6).
Requires specialized training	One participant (11%) felt that specialized training was necessary in order to properly probe questions in order to get an accurate assessment. This participant explained, “I think that without training in severe mental health disorders that maladaptive umm section of the Vineland doesn’t mean anything to anyone. You’ve gotta sort of read between the lines when they sit there and say, you know, do you are you afraid of everyday situations, EG elevators etcetera? You’ve gotta then understand that there are actually trying to key into a phobia and then you use that information to really dig deep. Because maybe moms, like. Oh yeah, they don’t like elevators. OK, cool. Why? And is it at the point where they won’t take one or they’ll jump in one. But you’ve got to have an iPad in front of them. OK, well, if they can do it, then it’s not a phobia” (PT4).

## Discussion

In our study, we sought to explore barriers encountered by clinicians during administration of the Bayley-4 and Vineland-3 to individuals with AS and offer guidance for their use. We identified challenges to administering both assessments related to characteristics of the assessor, individual with AS, and their caregiver; external factors like interruptions; organizational aspects such as timing; and challenges in determining the most appropriate answer. We also identified solutions for each challenge. For example, for the Bayley-4 barrier “lack of motivation”, participants suggested solutions including punctuality, engaging the parents, switching to a more engaging section, and keeping the examiner in the spotlight. Similarly, for the Vineland-3 barrier “determining start point”, participants recommended solutions such as reviewing previous Vineland administrations, basing the start point on observations of the participant, and starting at the beginning. Further, we examined the Bayley-4 and Vineland-3 items, revealing overarching concerns complicating their administration to individuals with AS.

A critical component of ensuring successful administration of these measures is adequate preparation on the part of the assessors [10]. Preparation includes considering test administration strategies such as building rapport, being prepared, and knowing the assessment items well through repetitive practice. Establishing trust and comfort can also enhance the assessment experience. Furthermore, thorough preparation (e.g., arriving early, setting up the assessment environment and materials in advance, familiarizing oneself with the assessment items) is essential for ensuring the efficiency and accuracy of the evaluation process. A comprehensive understanding of the assessment items is vital for navigating the assessment and accurately interpreting responses.

Administering the Bayley-4 assessment to individuals with AS presents unique challenges due to the assessment’s original development for children between 16 days and 42 months of age. Some of the older AS participants may find the manipulatives utilized in the Bayley-4 to be uninteresting or unengaging, and a lack of engagement may lead to an inaccurate representation of the individual’s developmental level [9]. Further, to address these challenges, assessors must employ strategies such as promptly meeting participants upon arrival at the clinic, escorting them directly to the assessment room, minimizing distractions in the assessment room to the extent possible, seamlessly transitioning between items, and motivating the participant with rewards (e.g., treats, breaks) and entertaining approaches. In addition, most individuals have severe to profound levels of ID and are predominantly nonspeaking. Flexibility in adapting the assessment approach to accommodate the individual’s communication abilities and interests is paramount for ensuring accurate evaluation outcomes in individuals with AS.

Administering the Vineland-3 to caregivers of individuals with AS presents distinct challenges. One of the primary difficulties involves obtaining an accurate interpretation of the capabilities of individuals with AS, as reported by their caregivers [34]. This task is complicated by the potential for differing opinions between caregivers regarding their loved one’s abilities. Assessors must navigate this challenge by facilitating open communication and collaboration between caregivers to ensure a cohesive and comprehensive assessment of the individual’s skills and functioning. Additionally, when conducting the Vineland-3 assessment virtually, assessors must be vigilant in minimizing distractions for the caregiver, who may be simultaneously engaged in daily tasks such as cooking or attending to other children. Furthermore, assessors should possess a deep familiarity with the Vineland-3 items to recognize spontaneous responses that may preemptively answer future questions, thereby avoiding redundancy and optimizing assessment efficiency. It is also essential to acknowledge that the items in the Maladaptive Behavior domain of the Vineland-3 may be inappropriate or irrelevant for individuals with AS, such as those pertaining to concepts like suicide. Assessors must exercise sensitivity and discretion in selecting and administering items to ensure the assessment accurately captures the individual’s abilities and needs while maintaining relevance and appropriateness for the AS population.

Lastly, assessors should work to build rapport and set expectations in order to address the possible emotional impact of the Vineland-3. Our interview participants warned that the progressive nature of Vineland-3 questions often leads caregivers to be increasingly aware of their loved one’s lack of developmental progress. Possible solutions included approaching the assessment with empathy and considering items covered previously. In these cases, assessors can preempt the assessment by explaining the purpose of the Vineland-3 along with some of its shortcomings and provide time for a debriefing at the end. Further, assessors should consider how items discussed earlier in the assessment might relate to later questions. Although it is still necessary to have the participant respond to a question, the assessor can, in these cases, refer to the previous conversations in order to provide context.

These barriers also highlight the importance of comprehensive training and standardized administration. Ensuring all assessors are uniformly trained in the nuances of administering the Bayley-4 and Vineland-3 to individuals with AS is critical for achieving consistent and accurate results. This includes not only didactic, lecture-based training, but also practical, hands-on experience and the opportunity to discuss and troubleshoot specific challenges with peers and mentors. Moreover, the development and implementation of standardized protocols can help mitigate the variability in assessment practices, leading to more reliable data collection. By focusing on thorough and consistent preparation, clinical trial sites can enhance the validity of the assessments and contribute to a deeper understanding of AS, ultimately improving care and intervention strategies. Beyond the clinical trial setting, insights from these data has the potential for broad practical implications for clinicians working with populations of individuals with AS, as many of these lessons can be implored in a clinical setting as best practice. These findings also help to aid in ongoing consultation and collaboration with assessment publishers in consideration of supplemental manual development and item level evaluations to identify acceptable accommodations that can be used without compromising validity or reliability of standard scores achieved in these individuals.

While no prior studies have systematically examined administration challenges for the Bayley-4 and Vineland-3 in individuals with Angelman syndrome, we are also unaware of published research that explores these challenges in other populations with intellectual disabilities. As such, our findings help address a broader gap in the literature, highlighting the need for syndrome-specific guidance on assessment use. These results may inform similar work in other neurodevelopmental populations where communication limitations and atypical developmental trajectories affect administration fidelity.

Future research should also explore caregiver perspectives on the use of these assessments, including their emotional responses, perceived relevance of test items, and overall burden of participating in the process. These perspectives will be essential for ensuring that assessments are not only psychometrically sound, but also ethically and practically feasible for families.

Our study has some limitations. It relies on secondhand accounts of assessors administering the Bayley-4 and Vineland-3 rather than direct observation. While multiple participants identified the same barriers and solutions, participants may report only salient factors—therefore, our results may lack comprehensiveness. Further, we report on small and racially homogenous group of assessors. Importantly, AS is a rare disease with a concomitant number of clinicians who administer the Bayley-4 and Vineland-3 across AS clinics worldwide and clinical trials. As such, it is reasonable to include a smaller number of interview participants, especially considering our varied geographic representation [35]. Still, future studies may aim to recruit a more racially diverse group of administrators if possible. Additionally, while this study identifies key barriers and solutions, it does not evaluate the feasibility or effectiveness of proposed strategies in practice. Future research should explore whether modifications to scoring criteria—such as accommodating AAC use in expressive language assessments—could improve validity. Similarly, integrating real-world observational data, such as caregiver-recorded video samples, may supplement standardized assessments and provide additional context for scoring.

## Conclusions

Our study provides important insights into shortcomings of the Bayley-4 and Vineland-3 when administered to individuals with AS. Many identified barriers reflect empirical observations based on clinical experience, such as engagement difficulties and the need for flexible administration when working with individuals with AS, even though these issues have not been systematically examined in published research. However, our study also revealed less recognized challenges, including caregiver emotional distress affecting Vineland-3 scoring and difficulty determining appropriate start points due to caregiver underestimation. Additionally, assessors noted that overexcitement—rather than disengagement—sometimes disrupted Bayley-4 testing. These findings highlight the need for tailored assessor training and caregiver support to improve administration feasibility.

Many upcoming clinical trials will rely on these as primary COAs, and the barriers and associated solutions discussed here can help inform their use and help improve the validity and reliability of these scores. These solutions provide a path forward for improving administration of the Bayley-4 and Vineland-3 to individuals with AS and other IDs in clinical practice, research studies, and clinical trials. While some of the identified solutions were shaped by individual clinician preferences and experiences, many offer broader guidance for professionals conducting assessments with individuals with AS. Certain strategies—such as adjusting the pacing of assessments, structuring testing environments to minimize distractions, and setting caregiver expectations—can be standardized across settings to improve assessment feasibility. Other solutions, such as engagement techniques or adapting test administration approaches, may require clinicians to personalize their methods while maintaining adherence to standardized protocols. These findings highlight the importance of training assessors to recognize common barriers and apply structured yet flexible strategies to improve assessment administration. Additionally, caregivers may benefit from clearer guidance on what to expect during assessments, which could help reduce emotional strain and enhance engagement in the process. Ultimately, by identifying key barriers and strategies to improve administration, this research can help enhance the validity of outcome measures in clinical trials while also making the assessment process more effective and supportive for clinicians and caregivers alike.

## Electronic Supplementary Material

Below is the link to the electronic supplementary material.

Supplementary Material 1

Supplementary Material 2

## Data Availability

The datasets used and/or analyzed during the current study are available from the corresponding author on reasonable request.
