# Supplementary material for: Using the Bayley-4 and Vineland-3 in Angelman syndrome: barriers, solutions, and challenging items
Source: Orphanet J Rare Dis. 2025 Jun 3;20:273. doi: 10.1186/s13023-025-03817-x (PMC12135291; doi:10.1186/s13023-025-03817-x)
Supplement: Supplementary file 1 — Supplementary Material 1 [file 13023_2025_3817_MOESM1_ESM.docx]

**Appendix A. Pre-Interview Survey**

**Demographic survey**

**Please complete this survey about yourself. We are asking you to provide general demographic information as well as information about your educational and training experiences.**

***General Demographic Information***

1. What is your current age? (field for individual to enter age in years)
2. What is your assigned sex at birth?
3. Male
4. Female
5. What gender do you identify as?
6. Female (she/her/hers)
7. Male (he/his/him)
8. They, them
9. None of the above
10. Do you live in the United States? (Y/N)
11. (If yes) Which state do you live in? (dropdown list of US states)
12. (If no) Which country do you live in? (field for individual to enter their country)
13. (If US-based) Please tell us about your race and ethnicity.

Which categories describe you? Please select all that apply. Note, you may select more than one group.

1. **American Indian or Alaska Native** (For example: Aztec, Blackfeet Tribe, Mayan, Navajo Nation, Native Village of Barrow Inupiat Traditional Government, Nome Eskimo Community, etc.)
2. **Asian** (For example: Asian Indian, Chinese, Filipino, Japanese, Korean, Vietnamese, etc.)
3. **Black, African American, or African** (For example: African American, Ethiopian, Haitian, Jamaican, Nigerian, Somali, etc.)
4. **Hispanic, Latino/a/e/x, or Spanish** (For example: Colombian, Cuban, Dominican, Mexican or Mexican American, Puerto Rican, Salvadoran, etc.)
5. **Middle Eastern or North African** (For example: Algerian, Egyptian, Iranian, Lebanese, Moroccan, Syrian, etc.)
6. **Native Hawaiian or Other Pacific Islander** (For example: Chamorro, Fijian, Marshallese, Native Hawaiian, Tongan, etc.)
7. **White** (For example: English, European, French, German, Irish, Italian, Polish, etc.)
8. **None of these fully describe me**
9. **Prefer not to answer**

***Education and Training Experience***

1. What is the highest level of education you have completed?
2. High school diploma or less
3. Some college
4. Bachelor’s degree
5. Master’s degree (Record type of degree __________________)
6. Doctoral degree (Record type of degree __________________)
7. Other
8. How many years have you spent working with people with neurogenetic/neurodevelopmental disabilities?
   1. In clinical/healthcare: ____________ years
   2. In clinical trials: ___________ years
   3. In non-clinical trial research: ___________ years
9. How many years have you spent researching individuals with Angelman syndrome?
   1. In clinical/healthcare: ____________ years
   2. In clinical trials: ___________ years
   3. In non-clinical trial research: ___________ years
10. What is the age range of the individuals with Angelman syndrome with whom you have worked?

***Bayley and Vineland Experience***

1. Which of the following assessments do you administer? (choose all that apply)
   1. Bayley Scales
   2. Vineland
2. What year did you first administer the Bayley Scales to any population? __________
3. How were you trained in administering the Bayley Scales for any population? (check all that apply)
   1. Observed videos of others performing the Bayley scales
   2. Reviewed the Bayley Scales manual
   3. Other- please list _____________
   4. No formal training
4. What year did you first administer the Bayley Scales for persons with AS? __________
5. In what contexts have you administered the Bayley Scales
   1. Clinical/Healthcare
   2. Clinical trials
   3. Non-clinical trial research
6. What is the estimated total number of times you have administered the Bayley Scales?
   1. In clinical/healthcare: ____________ times
   2. In clinical trials: ___________ times
   3. In non-clinical trial research: ___________ times
7. What versions of the Bayley Scales have you administered?
   1. Version 1
   2. Version 2
   3. Version 3
   4. Version 4 **[note- only participants who have administer the 4^th^ version will be eligible]**
8. What language do you administer the Bayley in to persons with AS?
   1. English
   2. Spanish
   3. Other: Please list ________
9. What section of the Bayley Scales are most challenging to administer? (Please note, we will ask more about each section during the interview)
   1. Cognitive Scale
   2. Language Scale- Receptive Communication Subset
   3. Language Scale- Expressive Communication Subset
   4. Motor- Fine Motor Subset
   5. Motor- Gross Motor Subset
   6. Behavioral Observation Inventory
10. What year did you first administer the Vineland? __________
11. In what contexts have you administered the Vineland
    1. Clinical/Healthcare
    2. Clinical trials
    3. Non-clinical trial research
12. What is the estimated total number of times you have administered the Vineland Scales?
    1. In clinical/healthcare: ____________ times
    2. In clinical trials: ___________ times
    3. In non-clinical trial research: ___________ times
13. What edition of the Vinland have you administered?
    1. 1^st^ Edition
    2. 2^nd^ Edition
    3. 3^rd^ Edition
14. How were you trained in administering the Vineland? (check all that apply)
    1. Observed videos of others performing the Vineland
    2. Reviewed the Vineland manual
    3. Other- please list _____________
    4. No formal training
15. What section of the Vineland are most challenging to administer? (Please note, we will ask more about each section during the interview)
    1. Communication Domain- Receptive
    2. Communication Domain- Expressive
    3. Communication Domain- Written
    4. Daily Living Skills Domain- Personal
    5. Daily Living Skills Domain- Domestic
    6. Daily Living Skills Domain- Community
    7. Socialization Domain- Interpersonal Relationships
    8. Socialization Domain- Play and Leisure
    9. Socialization Domain- Coping Skills
    10. Motor Skills Domain- Gross Motor
    11. Motor Skills Domain- Fine Motor
    12. Maladaptive Behavior Domain- Internalizing
    13. Maladaptive Behavior Domain- Externalizing
    14. Maladaptive Behavior Domain- Critical Items
