# Supplementary material for: Using the Bayley-4 and Vineland-3 in Angelman syndrome: barriers, solutions, and challenging items
Source: Orphanet J Rare Dis. 2025 Jun 3;20:273. doi: 10.1186/s13023-025-03817-x (PMC12135291; doi:10.1186/s13023-025-03817-x)
Supplement: Supplementary file 2 — Supplementary Material 2 [file 13023_2025_3817_MOESM2_ESM.docx]

**Appendix B. Interview Guide**

**Angelman Outcome Measure Training Study**

Interview Guide

*Note to the interviewer. Throughout this interview guide notes will be typed in this italicized blue font which are not meant to be read aloud to the participant. Rather they are instructions to help you move more smoothly through the guide. Please familiarize yourself with the guide before conducting the interview. Each section has an overarching question with a list of probes underneath. There is no need to ask a probing question if the participant has already provided that information when answering an earlier question.*

*The guide is organized with space between questions and in the margins so you will have room to jot notes as you go along. Please use these notes to help orient yourself toward questions that you want to ask later, and to write down your reflections.*

***Introduction***

Thank you for taking time to talk with me today. I am [name] from RTI International, an independent non-profit research institute based in North Carolina. We are working with the Angelman Biomarkers and Outcome Measures Alliance on a study to better understand administration of the Bayley Scales of Infant Development 4 and the Vineland Adaptive Behavior Scales, 3^rd^ edition, in research studies and clinical trials. The purpose of our interview is to help us understand challenges with administering these two instruments and develop guidance for future use.

This interview will take between 45 and 90 minutes of your time. In the first part of our interview, I will ask you some questions about your background and experience with Angelman syndrome more generally. Next, we will talk more specifically about the Bayley and Vineland—starting with questions about the Bayley including your background administering the Bayley and the setting where you typically would administer the Bayley. Finally, we will talk about any overall challenges you have experienced with administering the Bayley, along with some specific questions oriented toward different sections. Next, I will ask a similar series of questions about the Vineland. Some of the questions I ask may sound repetitive, or they may cover topics that you mentioned previously. Please know that I am listening carefully, and I want to ensure I have answers in your words.

I will use an audio recorder to record our conversation so that I can ensure I don’t miss anything you say. The audio recording will be transcribed and used for analysis. We will try our best not to use any identifying information, like names, during the recording, but if those things come up, they will be removed from the transcription. Only the study team will have access to the audio recording and transcript. Do I have your permission to audio record our interview?

*If No,* unfortunately we won’t be able to continue with the interview without the recording. That is okay, and I respect your decision and appreciate your time. *You can end the interview now*

*If Yes,* great! I will go ahead and start the audio recording now.

I have started the recording. I am talking with [Participant ID] for 0219237.000.001 and today’s date is [date].

Thank you again for talking with me today. Can you please verbally confirm again that you consented to be audio recorded during our interview?

*Wait for participant to answer yes.*

Thank you. I am going to ask you some questions that I would like you to answer to the best of your ability.

***Part A: Experience Information***

The first few questions are about your professional experience. I am asking this so we can describe your expertise.

1. To start, please briefly tell me about your experience working with people with genetic disorders, including Angelman syndrome.

*Probe:* What conditions? Especially those causing developmental delays.

*Probe:* What is the age range of the individuals with Angelman syndrome with whom you have worked?

*Probe:* Do you typically administer both the Bayley and the Vineland in the same day? And if so, which do you administer first? Why?

*Probe:* How do you handle the cognitive demands of administering the Bayley and Vineland? Across multiple participants the same day?

*Probe:* How are the Bayley and Vineland typically scheduled?

***Part B: Bayley Scales Experience***

Next, I am going to ask some questions about your experience with the Bayley Scales.

Please tell me about any training you have completed for administering the Bayley Scales, which versions? *Keep this in mind for Part C. later when we ask about administration of the Bayley.*

*Probe:* For the current clinical trial in AS- Quality techniques used to ensure consistent administration? Periodic trainings/audits?

***Part C: Bayley Administration Setting and Order***

*This section relates to administering the Bayley.* I would now like to talk about the typical setting(s) in which you administer the Bayley.

1. Please briefly describe what the child experiences when they first begin interacting with staff on the day of the Bayley administration and up until they have finished with all study activities.

*Probe:* How long has the participant been in the clinic? Time of day (and how that is taken into consideration)?

*Probe:* Briefly, other tasks the participant has engaged with in the clinic prior to your administration of the Bayley.

*Probe:* How much time do you typically have allotted to administer the Bayley? (Any time constraints either for the participant or administrator?)

*Probe:* How long does it typically take to administer the Bayley?

*Probe:* Breaks given during/before administration?

*Probe:* Do you ever re-administer items for any reason (for example, item answers the child didn’t respond to)? If so, please explain why. How do you keep track of what you need to go back to re-administer?

1. Are there other individuals in the room? If so, who?

*Probe:* Do they have a parent/caregiver with them during administration? If not, where is the parent/caregiver located.

*Probe:* Are there any other people (non-parents/caregivers) in the room?

1. *if clinician and researcher-* Are there any differences to how you administer the Bayley in a clinical setting versus in a research study?

*Note to interviewer, question number 4 should only be asked if the participant indicated that others are in the room during time of the test administration.*

1. Does the participant ever interact with the other individuals in the room while you are administering the Bayley?

*Probe:* Do you provide guidance to the caregiver on ways they should or should not engage with you or the child while the Bayley is being administered? If so, what do you advice?

*Probe:* In what situations do caregivers or others in the room communicate either verbally or nonverbally? *We especially want to know whether others in the room are giving cues, whether it is purposeful or not.*

*Probe:* How do you handle any type of communication from the caregiver whether verbal (e.g., talking, vocalizations) or non-verbal (e.g., eye contact, gestures)?

1. Please describe the space (or spaces) where you have typically administered the Bayley.

*Probe:* Number of different rooms used to administer the tests to a single individual? ____________

*Probe:* Are any other procedures or surveys administered to the patient in the same room as the Bayley (e.g. blood draws, etc.)? If so, are any of these done prior to the Bayley administration.

*Probe:* Physical environment description (e.g., size, observation window/one way mirror, windows, doors, lights, sounds—can you hear others through the wall?)

*Probe:* Furniture in the room and orientation of that furniture

*Probe:* Any toys, materials, that are present and may be distracting during the assessment. (If so, how are these handled?); are the materials required for the Bayley stored until they are needed?

*Probe:* How is the person with AS oriented in the room? (type of chair they are seated in, orientation with others).

*Probe:* If there are other people in the room, how are they oriented? (e.g., is the parent sitting right next to the participant with AS and facing the administrator? Is there a desk separating the administrator and the participant? Where does the administrator sit in relation to the participant during each test?)

***Part D: Bayley Overall Challenges***

In the next section I want to hear from you more specifically about any potential challenges to administering the Bayley.

1. First, have you encountered any behavioral challenges from your typical participant with AS that impact how these tests are administered? What do you typically do to motivate the participant?

*Probe:* What do you do to motivate the participants?

*Probe:* What are the challenges in motivation? (Do you engage with the family for ways to overcome the challenges in motivation?)

*Probe:* Strategies for achieving compliance (e.g., incorporation of augmentative and alternative communication (AAC) devices, getting ideas/materials/snacks from parents/caregivers; use of reinforcers such as M&M’s or Goldfish crackers)

*Probe:* How do you account for situations that might impact their performance (e.g., travel, staying in hotels, a recent illness)?

1. Please tell me about how you go determining whether the administration of the Bayley was valid.

*Probe:* How do you determine if the participant doesn’t know something versus it being a behavior/compliance issue?

*Probe:* Do you ever ask the parent/caregiver for feedback on the validity of answers?

*Probe:* What do you do when the parent reports the child can perform certain tasks that are not directly observed?

1. Have you ever had to stop the administration of either the entire Bayley or sections of the Bayley? If so, please describe that situation. If you have, have you had the opportunity to readminister it?
2. Next, I want to think about the frequency at which the administrations are done. What is the shortest interval between administrations you think allows for reliable and valid scores?

*Probe:* Disadvantages of more frequent administration?

*Probe:* Do you ever change the frequency of administering the Bayley based on results from previous administrations?

*Probe:* How do you handle it if there are different caregivers present at the different administrations?

***Part E: Bayley Administration***

We are almost done. Now I want to ask some specific questions about the Bayley. I am going to show you a Bayley-4 protocol. In a moment we will briefly walk through each section of the Bayley, but first I will have some general questions for you. *Share the version of the Bayley Scales the participant identified previously that they used during the clinical trial.*

1. What order do you typically administer the different domains of the Bayley to individuals with Angelman syndrome?
2. How do you determine what point to start the Bayley? (Ask for new patient and repeated administrations).

*Probe:* Based on previous tests? For repeat administrations how do you determine start points?

1. What domains or items of the Bayley are the **most difficult** for you to administer to individuals with Angelman syndrome?
2. What domains or items of the Bayley are the **easiest** for you to administer to individuals with Angelman syndrome?
3. Next, I am going to ask you to briefly reflect on any difficulties you have experienced administering specific items for each of the domains to individuals with Angelman syndrome. For each of the domains, what items are most challenging to administer?

*Scroll through the Bayley to show the participant each domain. Here is a link to the instrument🡪* [BSID-4 Record Form.pdf](https://researchtriangleinstitute.sharepoint.com/:b:/r/sites/AngelmanOutcomeMeasureTraining/Shared%20Documents/General/Record%20forms/BSID-4%20Record%20Form.pdf?csf=1&web=1&e=hqhXju)

*Probe:* Cognitive Scale

*Probe:* What works for hiding tasks? (e.g., does assessor use a block or another more motivating object for the item?)

*Probe:* Language Scale- Receptive Communication Subset

*Probe:* Language Scale- Expressive Communication Subset

*Probe:* Do you base your scores on observation, parent report, or a combination of both? Challenges with this?

*Probe:* Motor- Fine Motor Subset

*Probe:* Motor- Gross Motor Subset

*Probe:* Behavioral Observation Inventory

***Part F: Bayley Scales Experience***

This is the final section on the Bayley before we move on to the Vineland.

1. If you were training someone new on how to administer the Bayley, what would you recommend to help make their administrations as smooth as possible?

***Part G: Vineland Experience***

Next, I am going to ask you some questions about your experience with the Vineland.

1. Please tell me about any you received for conducting the Vineland? Which versions?

*Probe:* What quality techniques have you experienced to ensure consistent administration? (e.g., Periodic trainings/audits)

***Part H: Vineland Administration Setting and Order***

*Please note whether the participant indicated the Vineland is administered on the same day as the Bayley previously.* I would now like to talk about the setting(s) in which you administer the Vineland.

1. In general, please describe administration of the Vineland.

*Probe:* Is there more than one person you are talking with over the phone?

*Probe:* How long does it take to administer the Vineland?

*Probe:* Breaks given during/before administration?

*Probe:* Do you ever clarify responses for any reason (for example, item answers you are unclear about)? If so, please explain why.

1. What modality do you typically use to administer the Vineland (i.e., in-person, by-phone, video-teleconferencing)?
2. *If administered by phone or video-teleconferencing-* Please briefly describe the process of administrating the Vineland by phone or video-teleconferencing.
3. *If administered in-person-* Are there other individuals in the room? If so, who?

*Probe:* Do they have the child with them during administration? If not, where is the child located.

1. *If clinician and researcher-* Are there any differences to how you administer the Vineland in a clinical setting versus in a research study?

***Part I: Vineland Overall Challenges***

In the next section I want to hear from you more specifically about any potential challenges to administering the Vineland.

1. Have you ever had to stop the administration of the Vineland (e.g., scheduling, emergency)? If so, please describe that situation.
2. Next, I want to think about administering the Vineland at different time points. What is the shortest interval between administrations you think allows for reliable and valid scores?

*Probe:* Disadvantages of more frequent administration?

***Part J: Vineland Administration***

Now I want to ask some specific questions about the Vineland. I am going to show you a protocol of the Vineland-3. In a moment we will briefly walk through each section of the Vineland, but first I will have some general questions for you.

1. Do you ever have a change in the caregiver who is acting as the reporter across different administration timepoints? If so, how do you handle this?
2. How do you determine where to start the Vineland?

*Probe:* For repeat administration how do you decide the start point?

*Probe:* Do you have access to previous administrations? If so, do you ever review the previous administrations before starting a new one?

1. Do you conduct the assessment online via Q-Global or using a paper pencil format using the booklet?
2. If the caregiver is using the caregiver report form version of the Vineland (where the caregiver completes the Vineland questionnaire on paper themselves) instead of completing the comprehensive interview with you, do you review the answers with the caregiver?
3. How do you determine 1 and 2 scoring when discussing items with a caregiver?
4. How do you deal with discrepancies between what you as a rater know to be true and what a parent is telling you?
5. Do you ever look back at previously completed Vineland assessments to see how the questions were answered (e.g., to help position how answers should be marked in the current administration)?
6. Do you administer the Maladaptive Behavior domain?
7. What domains or items of the Vineland are the **most difficult** for you to administer to caregivers of individuals with Angelman syndrome? Why?

*Probe:* Challenges in achieving basal/ceiling?

1. What domains or items of the Vineland are the **easiest** for you to administer to caregivers of Angelman syndrome? Why?
2. Next, I am going to ask you to briefly reflect on any difficulties you have experienced administering each of the domains to caregivers of Angelman syndrome. For each of the domains, which items are particularly challenging for you to administer, more open to caregiver interpretation, or harder for you to interpret?

*Scroll through the Vineland to show the participant each domain. Here is a link to the instrument🡪* [Vineland-3 Comprehensive Interview Record Form.pdf](https://researchtriangleinstitute.sharepoint.com/:b:/r/sites/AngelmanOutcomeMeasureTraining/Shared%20Documents/General/Record%20forms/Vineland-3%20Comprehensive%20Interview%20Record%20Form.pdf?csf=1&web=1&e=yyT6E5)

*Probe:* Communication Domain- Receptive

*Probe:* Communication Domain- Expressive

*Probe:* Communication Domain- Written

*Probe:* Daily Living Skills Domain- Personal

*Probe:* Daily Living Skills Domain- Domestic

*Probe:* Daily Living Skills Domain- Community

*Probe:* Socialization Domain- Interpersonal Relationships

*Probe:* Socialization Domain- Play and Leisure

*Probe:* Socialization Domain- Coping Skills

*Probe:* Motor Skills Domain- Gross Motor

*Probe:* Motor Skills Domain- Fine Motor

*Probe:* Maladaptive Behavior Domain- Internalizing

*Probe:* Maladaptive Behavior Domain- Externalizing

*Probe:* Maladaptive Behavior Domain- Critical Items

***Part K: Vineland Scales Experience***

This is the final section on the Vineland.

1. If you were training someone new on how to administer the Vineland, what would you recommend to make their administrations as smooth as possible?

***Part L: Closing***

Thank you for your feedback. The answers you gave will help us to better understand how these two tests are administered. Are there any other important aspects that we have not discussed that are relevant to our aim?

Thank you for your time!
